# The Role of Music in Everyday Life During the First Wave of the Coronavirus Pandemic: A Mixed-Methods Exploratory Study

**DOI:** 10.3389/fpsyg.2021.647756

**Published:** 2021-05-04

**Authors:** Emily Carlson, Johanna Wilson, Margarida Baltazar, Deniz Duman, Henna-Riikka Peltola, Petri Toiviainen, Suvi Saarikallio

**Affiliations:** Department of Music, Arts and Culture Studies, Centre for Interdisciplinary Music Research, University of Jyväskylä, Jyväskylä, Finland

**Keywords:** music, coronavirus, music listening, anxiety, emotion regulation

## Abstract

Although music is known to be a part of everyday life and a resource for mood and emotion management, everyday life has changed significantly for many due to the global coronavirus pandemic, making the role of music in everyday life less certain. An online survey in which participants responded to Likert scale questions as well as providing free text responses was used to explore how participants were engaging with music during the first wave of the pandemic, whether and how they were using music for mood regulation, and how their engagement with music related to their experiences of worry and anxiety resulting from the pandemic. Results indicated that, for the majority of participants, while many felt their use of music had changed since the beginning of the pandemic, the amount of their music listening behaviors were either unaffected by the pandemic or increased. This was especially true of listening to self-selected music and watching live streamed concerts. Analysis revealed correlations between participants’ use of mood for music regulation, their musical engagement, and their levels of anxiety and worry. A small number of participants described having negative emotional responses to music, the majority of whom also reported severe levels of anxiety.

## Introduction

On March 11, 2020, the World Health Organize declared a global pandemic due to the spread of COVID-19, an infectious disease caused by a novel strain of coronavirus which was first identified in China near the end of the previous year. A notable feature of COVID-19 is pre-symptomatic and asymptomatic transmission, such that individuals could unknowingly pass the virus on to others while feeling healthy, leading to uncertainty about the situation for individuals and governments alike (Furukawa et al., 2020). In response to the pandemic, many countries instituted lockdown measures, closing non-essential businesses and schools, and cautioning citizens to stay at home, in order to slow the spread of the virus. Such measures have proven to effective when strictly implemented (Alfano and Ercolano, 2020; Sjödin et al., 2020), but for many countries has come at high cost to economic and social wellbeing (Ahmad et al., 2020; Herat, 2020; Vinkers et al., 2020). Widespread social isolation in conjunction with widespread access to the internet and other technology has created an unprecedented situation in many spheres of public and private life.

Public mental health has been raised as an important issue to address through research and practice during the coronavirus pandemic (Xiang et al., 2020). The European College of Neuropsychopharmacology has noted that while emotion distress and anxiety are normal responses to an unpredictable and threatening situation such as the pandemic (Vinkers et al., 2020), research suggests that rates of anxiety and depression have notably increased during the pandemic (Salari et al., 2020), with one study suggesting a rate of anxiety three times higher than pre-pandemic in Austria (Pieh et al., 2020). The mental health effect of the pandemic may be exacerbated by the necessity to remain in social isolation to prevent the spread of the virus (White and Van Der Boor, 2020). Not only has the coronavirus pandemic created an increased need for mental health support in many populations, it has complicated the delivery of professional mental health services, due to the need to rely on technology such as video conferencing (Ojha and Syed, 2020). It is therefore imperative to understand the strategies individuals use to cope with the negative psychological consequences of the pandemic, in order to support resilience on individual and community levels (Vinkers et al., 2020).

Just days after the declaration of a pandemic, a poignant image of life in lockdown reached many via various news outlets: Italian musicians playing and singing music from their balconies as a way to create a “moment of joy in this moment of anxiety” (Horowitz, 2020). A broad swath of research supports the use of music as a means of managing negative affect such as stress, anxiety or depression. Music-listening has been shown to affect the autonomic nervous system (Thoma et al., 2013; Harada et al., 2017) and to decrease state-anxiety (Gillen et al., 2008; Thoma et al., 2015), while meta-analysis has found that music-based interventions are effective in reducing human stress responses (Baltazar et al., 2019; de Witte et al., 2020). Music can play a role in important psychological functions such as identity construction (North and Hargreaves, 1999), mood and emotion regulation (Saarikallio, 2008; Carlson et al., 2015), personal agency and social competence (Saarikallio, 2019; Saarikallio et al., 2020). Music listening has been shown to be among the most common strategies individuals use when consciously attempting to improve their current affective state (Thayer et al., 1994). Playing or dancing to music together additionally fosters social connection (Keeler et al., 2015; Tarr et al., 2016).

In addition to the balcony performances given by Italians under lockdown, the first months of the pandemic were marked by a number of organizations, including the British Royal Opera House, the New York Metropolitan Opera and the New York Philharmonic, provided free streaming of performances (Barone, 2020). Artists including Yo-Yo- Ma and the Indigo Girls shared performances via social media using the hashtag #Songsofcomfort (Barajas, 2020). Such behaviors indicate a public perception that music and other performing arts may function to provide solace and comfort during times of national or international crisis. However, the unprecedented nature of the coronavirus pandemic raises important questions about the role of music in daily life, particularly as a tool for coping with the crisis. Many of the functions of music are social, and some argue that music is fundamentally social, communicative and embodied (Cross, 2009), which aspects are almost certainly altered for many by the pandemic situation. It is unknown whether the pandemic has caused changes in private listening behavior, whether increased stress anxiety related to the pandemic can be mitigated by music use, and whether some music uses and listening strategies are more effective than others in supporting mental health. It could be hypothesized that increased incidence of negative affect might cause more people to turn to music as a means to regulate mood and affect, but it may also be that increased stress and anxiety prevent individuals from using music effectively. Similarly, although participation in and attendance of live musical performance will certainly have changed due to lockdown measures, it is unknown whether this affects other behaviors of engaging with music, such as listening to recordings or watching live-streamed or recorded performances, and whether these changes have an important impact on the functions of music.

Understanding how music is (or is not) being used by individuals for affect regulation during the coronavirus pandemic is an important step to understand whether and how music could be used deliberately to mitigate negative effects of lockdown and prolonged stress and uncertainty. The current study aims to contribute to the development of this understanding by addressing the following research questions:

1) Has the coronavirus pandemic had an influence on individuals’ musical engagement, and if so, how?
2) How are individuals engaging with music during the coronavirus pandemic?
3) How does this musical engagement relate to negative psychological effects of the pandemic, specifically worry and anxiety?

## Materials and Methods

Based on these research questions, an online survey was designed to collect demographic, situational, psychological, and music-specific data from participants. The survey consisted of a combination of previously existing measures, measures adapted from pre-existing measures, and measures developed specifically for the current study. Due to the highly exploratory nature of the research, the survey was designed to cast a wide net, and therefore includes more measures than have been selected for analysis in the current study.

### Measures

The Beck Anxiety Index (BAI) was chosen to measure participants’ anxiety levels. First proposed by Beck, Brown, Beck et al. (1988), the inventory consists of 21 items comprising both physical and cognitive symptoms of anxiety such as “Dizzy or lightheaded” or “Fear of losing control.” Participants rate on a four-point Likert scale indicating how much these symptoms have bothered them during the previous month. The BAI has been shown to have high reliability and validity for non-clinical as well as clinical populations (Creamer et al., 1995).

At the time of survey development, no known measures specific to COVID-19 had been developed. However, Cheng, Wong, Tsang, and Wong (Cheng et al., 2004) developed the SARS Impact Scale (SIS), a 12-item Likert-scale measure of psychological distress in survivors of the SARS, a novel coronavirus which caused a highly contagious outbreak in China and Hong Kong in 2002–2003, which included items such as ‘I will be killed by SARS’ and ‘I will pass the SARS virus onto my family.’ This survey, along with publicly available information about COVID-19 available from the World Health Organization, was used to develop a similar survey specific to people living during the COVID-19 pandemic. It included nine items, several of which were identical or nearly identical to items found in the SIS, such as ‘I worry I will lose my job and have financial problems because of coronavirus,’ and ‘I worry that I will pass coronavirus onto my family,’ as well as items specific to the COVID-19 situation at the time of survey-development, such as, ‘I worry that I have coronavirus and don’t know it.’

The Brief Music in Mood Regulation Questionnaire (B-MMR) was used to assess participants’ current use of music as a tool for affect regulation. The B-MMR is a 21-item scale developed by Saarikallio (2012) from an original 40-item MMR scale (Saarikallio, 2008), and assesses participants’ use of music for affect regulation by seven strategies: (1) *Entertainment*, which refers to using music to create a pleasant atmosphere or to make boring tasks more enjoyable, (2) *Revival*, in which music is used to feel refreshed and to gain energy when stressed or tired, (3) *Strong Sensation*, in which music is used to stimulate intense emotional and esthetic experiences, (4) *Diversion*, which refers to using music to distract from unwanted thoughts and feelings (5) *Discharge*, in which music is used to express and release of negative emotions, (6) *Mental Work*, in which music is used to support mental contemplation and clarification of emotional preoccupations, and (7) *Solace*, which refers to using music to gain feelings of comfort, acceptance, and understanding as a response to negative emotion. Each item is assessed using three 5-point Likert-scale items, such as ‘I listen to music to perk up after a rough day’ and ‘For me, music is a way to forget about my worries.’

Participants were asked whether they felt that the coronavirus pandemic had changed their everyday music use. If so, they were asked to rate how much it had changed using a 7-point Likert scale. To assess their engagement with music, participants were asked about the frequency of various music-listening and music-making behaviors using a 5-point Likert scale ranging from ‘Not at all’ to ‘Multiple times per week.’ The items assessed were: (1) Listened to music that I selected, (2) Listened to music that someone else selected, (3) Listened to music on the radio (or streaming), (4) Danced to music, (5) Watched a professional performance live (virtually), (6) Watched an amateur performance live (virtually), (7) Watched a pre-recorded professional performance, (8) Watched a pre-recorded amateur performance, (9) Watched music videos. If participants answered a question positively, they were subsequently asked with whom they had listened (if anyone), and given a free text-box to optionally provide more details in response to the question ‘Tell us more about this experience.’ Participants who indicated that they had actively made music as well as listened to music were asked to report whether they engaged in (1) Singing, (2) Playing an instrument, (3) Making music electronically, (4) Receiving or giving music lessons online. Participants were additionally asked whether their music listening had changed due to the pandemic, to rate on a 5-point scale how much their music listening had changed.

In addition to these measures, participants were asked to report which virtual platforms they used to engage with music (e.g., YouTube, Spotify), and whether their engagement with these platforms had changed. They were also asked to report perceived changes in their musical engagement for mood regulation since the start of the pandemic. These items are not included in the current study and will be analyzed at a later date.

### Procedure

The survey was administered using Alchemer^1^. Participants were informed via an introduction page the scope and intent of the research, that their data would be kept private and used anonymous, and that they were free to withdraw their consent to participate at any time. Participants were required to give their consent before moving on to the rest of the survey. The data under current analysis was gathered between April 2020 and June 2020. Data were analyzed using SPSS (v. 27), Matlab (v. 2018b), and Nvivo (v. 11 and 12).

## Results

### Participants

Participants were recruited using social media posts, University and professional e-mail lists, and via both English- and Finnish-language press releases. A total of 432 participants (238 identified as female, 175 male, 2 transgender and 13 non-binary) between the ages of 18 and 77 (M = 39.5, SD = 12.97) completed the survey. They comprised 37 nationalities, the largest cohort of which were citizens of Finland (35.1%), followed by Canada (18.5%) the United States (14.3%). A majority of participants (53%) reported being married or in a committed relationship, followed by participants who reported being single (36%), with the remainder being separated, divorced or widowed (11.2%).

Due to the conditions of lockdown in many countries, the living situation of participants was considered important to assess. The majority (69.3%) of participants reported living with others as opposed to living alone (30.7%). Of these, 53.1% reported living with a partner, 27% reported living with children, 12.9% reported living with siblings, 1.4% reported living with a grandparent or elderly relative, and 5.5% reported living with flatmates or friends; 13% reported living with at least one person with a disability or chronic illness. A minority (17.1%) reported that who they lived with had changed as a result of the pandemic. Most participants reported having virtual contact with immediate family between once and a few times per week (65.8%), somewhat fewer reported this with extended family (47.6%) and work colleagues (49.9%), and somewhat more with friends (69.3%).

**FIGURE 1:**
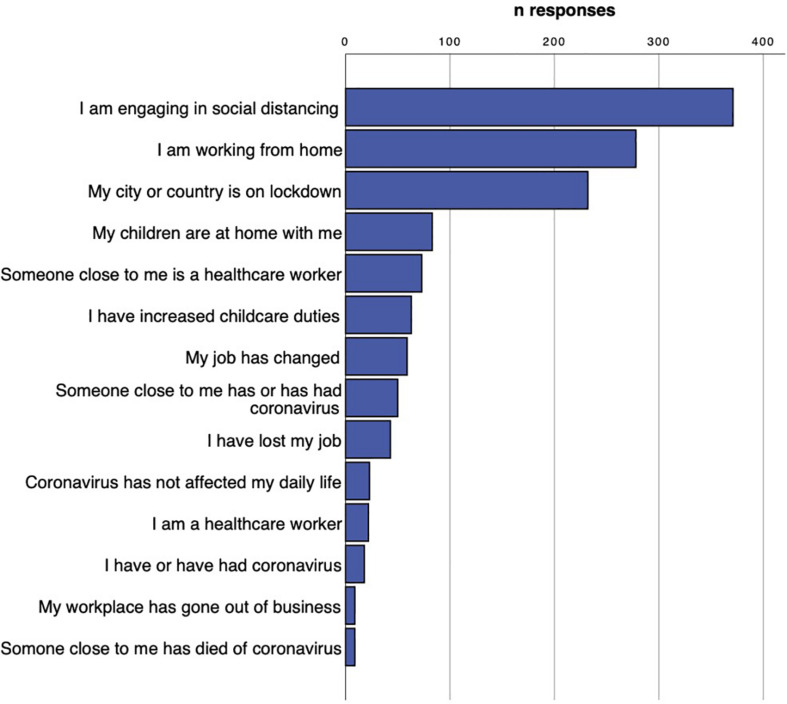
Reported daily living situation of participants as relates to the coronavirus pandemic.

Participants reported that the coronavirus pandemic had affected their daily lives in a number of ways, as shown in Figure 1. A notable majority (85.7%) reported that they are engaging in social distancing, and more than half (53.6%) reported that their city or country was on lockdown. A majority (64.2%) reported that they were working from home. A total of 9.9% reported having lost their jobs due to the pandemic, 4.2% reported having had the virus and 11.5% reported knowing someone who has had the virus. It should be noted that this data was gathered during the first half of 2020, during the first wave, and may not accurately reflect later months of the pandemic.

**FIGURE 2:**
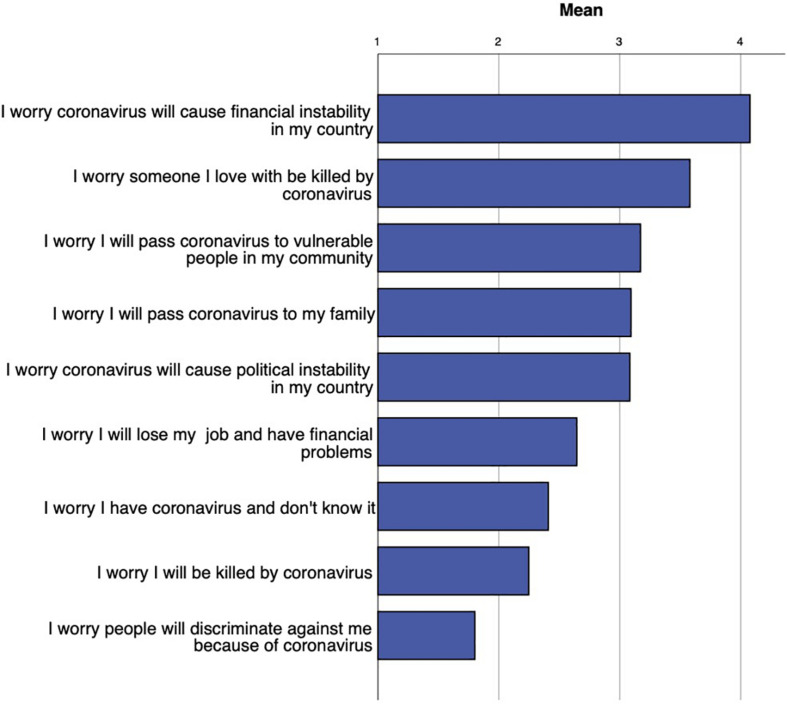
Coronavirus-related worries as reported by participants, ranging from not worried at all (0) to very worried (5).

Participants reported having worries that were specific to the coronavirus pandemic, as shown in Figure 2, with the highest rated worry relating to financial instability in participants’ countries, as well as worry that coronavirus may kill a loved one.

### Music Engagement Behaviors

**FIGURE 3:**
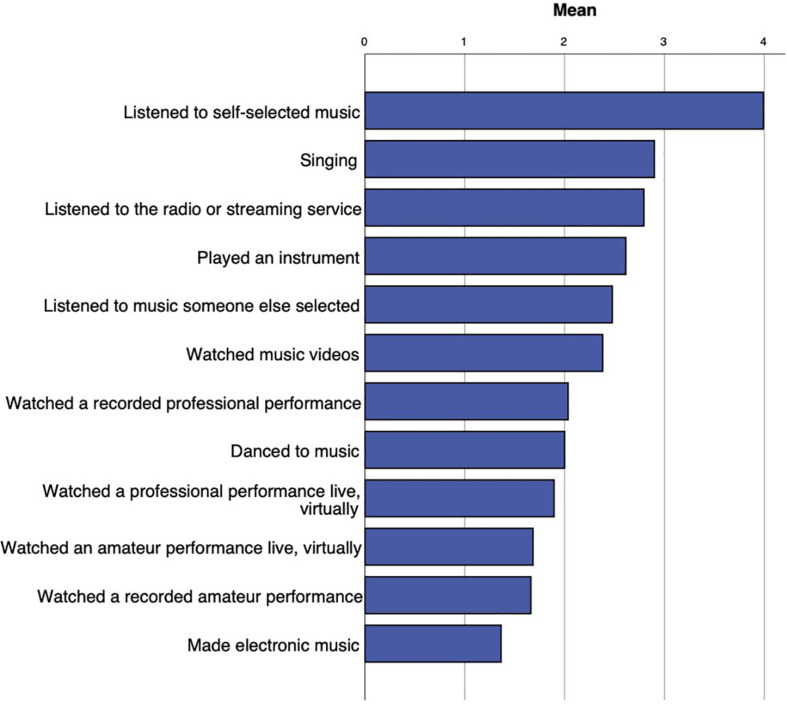
Participants’ ratings of how frequently they engaged in particular music listening behaviors, ranging from not at all (0) to multiple times per day (5).

More than half of participants (60.7%) reported that their use of music in daily life had changed as a result of the coronavirus pandemic. Within these participants, the perceived amount of change, rated on a 7-point Likert scale, showed an approximate normal distribution (*M* = 4.17, *SD* = 1.5), suggesting individual variation. Mean reported frequency of music behaviors is shown in Figure 3. The most frequently reported behavior was listening to self-selected music, with 37.6% of participants reporting listening to music multiple times per day. Slightly more than half (*n* = 228), reported that they had made music themselves. Of these, 86% reported singing at least once during the last 2 weeks, while 78% reported having played an instrument, and 24% reported having made music electronically.

Participants scores for each of the MMR strategies were all negatively skewed but none significantly so (skewness ranged from −0.037 to −1.23), suggesting that the current sample may have a slightly stronger tendency to use music in mood regulation than a randomly selected sample. This may be due to participants being more likely to complete the survey if they had an interest in music.

**FIGURE 4:**
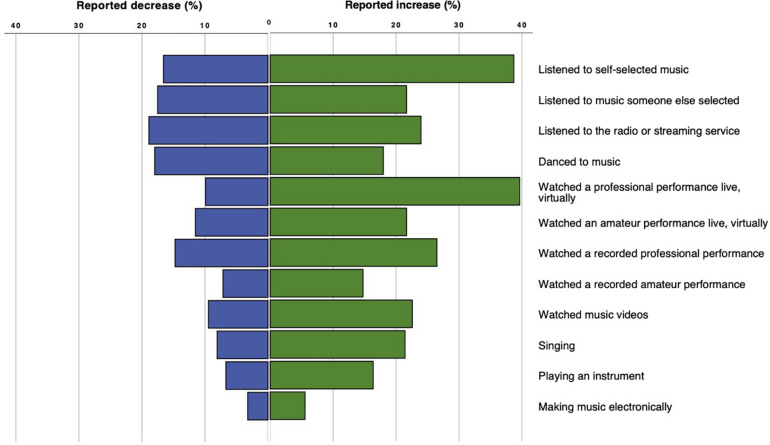
Percentage of participants who reported increases or decreases in a given behavior, of all who reported engaging in the behavior (n ranged from 38 to 432).

While participants reported both increases and decreases in these music engagement behaviors, the only behavior in which a majority of participants reported a change in the frequency of their behavior since the beginning of the pandemic was listening to self-selected music, for which only 44.7% of participants reported a change. Figure 4 show the percentage of participants who reported increases in various musical engagement behaviors respectively, of the participants who reported a given behavior (ranging from *n* = 432 for listening to self-selected music, to *n* = 38 for making electronic music).

### Relationships Between Music Variables, Anxiety, and Worry

To reduce the number of variables for further analysis, Principal Component Analysis (PCA) was performed on participants’ answers to several sections of the survey, specifically the COVID-19-Specific Worries measure, participants’ reported levels of engagement with various music-listening and music-making behaviors, and participants’ use of music to regulate mood via their B-MMR scores. After visual examination of scree plots, components which accounted for less than 9% of variance were discarded from further analysis.

**FIGURE 5:**
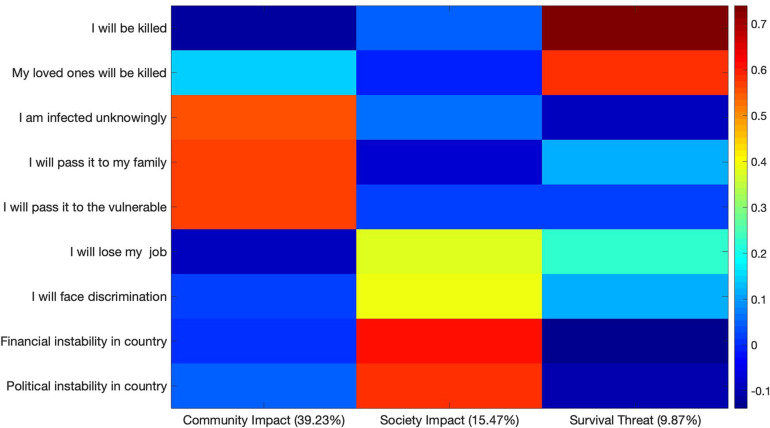
Varimax-rotated PCA solution for the COVID-Specific Worries. Three components account for a collective 64.57% of the variance.

The varimax rotated PCA solution for nine questions related to COVID-19-Specific Worries revealed a three-component solution accounting for 64.57% of variance, which is shown in Figure 5. The first component (PC1) accounted for 39.23% of the variance and included high loadings for the questions “I worry that I am infected with coronavirus and don’t know it,” and “I worry I will pass coronavirus onto my family,” and “I worry that I will pass coronavirus on to the vulnerable in my community.” PC1 was labeled Contagion Impact. A second component (PC2) accounted for 15.47% of the variance and included high loadings for the questions, “I worry that coronavirus will result in long-term financial instability in my country,” and “I worry that coronavirus will result in long-term political instability in my country,” as well as moderate loadings for the questions, “I worry I will lose my job because of coronavirus” and “I worry others will discriminate against me because of coronavirus.” PC2 was therefore labeled Societal Impact. A third component (PC3) accounted for an additional 9.87% of the variance, and included high loadings for the questions, “I worry that I will be killed by coronavirus” and “I worry that a loved one will be killed by coronavirus.” PC3 was labeled Survival Threat. The second two factors are loosely comparable to the first and third factors documented by Cheng et al. (2004) in the SIS and have been named similarly.

**FIGURE 6:**
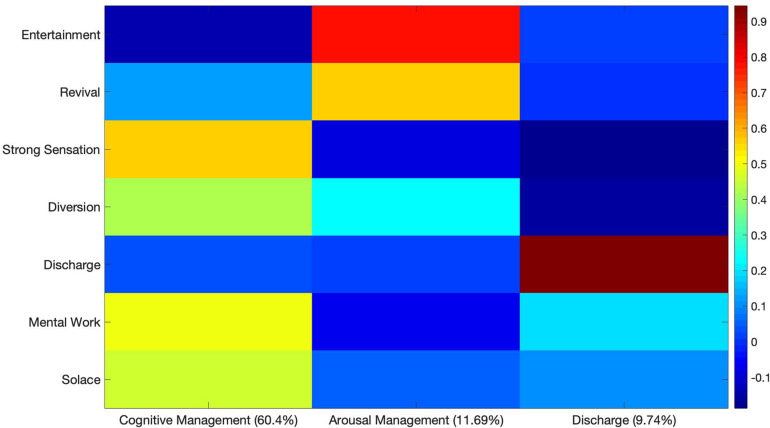
Varimax-rotated PCA solution for participants musical-engagement. Four components account for a collective 61.94% of the variance.

To examine participants’ musical-engagement, a PCA was performed including answers from participants indicating their current levels of engagement with a number of music listening and music playing behaviors. A four-factor solution, accounting for a total of 61.94% variance, was subjected to varimax rotation, shown in Figure 6.

The first component (PC1) accounted for 25.45% of the variance and included high loadings for listening to professional and amateur performances live, and moderate loadings for listening to professional and amateur performances which had been pre-recorded. PC1 was therefore labeled Music Performances. A second component (PC2) accounted for 14.29% of the variance, and included high loadings for singing or playing an instrument, and was therefore labeled Music Making. A third component (PC3) accounted for 12.16% of the variance, and included high loadings for listening to music selected by others and listening to radio or streaming, and a moderately high loading for dancing to music. PC3 was labeled External Music, referring music-listening in which the listener does not actively choose as well as the external behavior of dancing. A final component (PC4) accounted for an additional 10.04% of the variance, and included high loadings for listening to self-selected music and watching music videos. PC4 was labeled Chosen Music.

**FIGURE 7:**
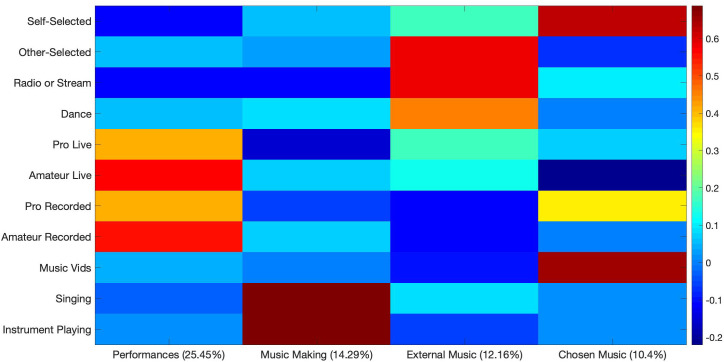
Varimax-rotated PCA solution for MMR scores. Three components account for a collective 81.83% of the variance.

A final PCA was performed on participants’ B-MMR scores, to assess patterns in their use of music as a coping mechanism. The results are displayed in Figure 7. A varimax-rotated three-component solution accounted for a total of 81.83% of variance. The first component (PC1) accounted for 60.4% of variance, and included moderately high loadings on Strong Sensation, Diversion, Mental Work and Solace. PC1 was labeled Cognitive Management, as Diversion involves the use of music to distract from thought and Mental Work entails the use of music to aid thinking. A second component (PC2) accounted for an additional 11.69% of the variance, and included a high loading for Entertainment and a moderately high loading for Revival. PC2 was labeled Arousal Management, as the need to relieve boredom as well as to feel energized can be considered arousal-related functions (Merrifield and Danckert, 2014). A final component accounted for 9.74% of the variance, and included a high loading for Discharge. PC3 was therefore simply labeled Discharge.

The relationships between participants’ PC scores regarding coronavirus worries, musical engagement, and uses of music for mood regulation was further explored. Correlation analysis revealed a number of small to medium significant correlations. To reduce the chance of Type-I error due to the number of comparisons, a Bonferroni corrected alpha level of.0004 was used as a threshold for significance. Results are shown in Table 1.

**TABLE 1:** Correlation of PC scores.

	**1**	**2**	**3**	**4**	**5**	**6**	**7**	**8**	**9**	**10**
1. Anxiety (BAI)	–									
2. Contagion Impact^1^	0.39*	–								
3. Societal Impact^1^	–0.00	0.00	–							
4. Survival Threat^1^	0.09	0.00	0.00	–						
5. Music^2^ Performances	0.01	0.12	–0.01	–0.06	–					
6. Music Making^2^	0.01	–02	–0.12	–0.14	0.00	–				
7. External Music^2^	0.12	0.12	–0.09	–0.04	0.00	–0.00	–			
8. Chosen Music^2^	–0.01	–0.09	–0.00	–0.02	0.00	–0.00	0.00	–		
9. Cognitive Management^3^	0.18*	0.27*	–0.02	–0.03	0.35*	−0.18*	0.21*	0.15	–	
10. Arousal Management^3^	−0.18*	–0.10	0.04	0.00	–0.01	–0.15	0.20*	–0.04	–0.00	–
11. Discharge^3^	0.12	–0.00	–0.02	0.01	−0.17*	–0.14	0.02	0.04	0.00	–0.00

*p < 0.0004. ^1^Covid worry PCs. ^2^Musical engagement PCs. ^3^Mood regulation PCs.

The strongest relationship was between Beck Anxiety Index (BAI) scores and participants and Community Impact, that is, worries regarding contagion of coronavirus (*r* = 0.39, *p* < 0.001). There were significant positive correlations between Cognitive Management and Anxiety (BAI) and, Contagion Worry (*r* = 0.18, *p* < 0.001), Musical Performances (*r* = 0.35, *p* < 0.001), External Music (*r* = 0.21, *p* < 0.001), and a significant negative correlation between Cognitive Management and Music Making (*r* = −0.18, *p* < 0.001). Arousal Management was significantly negatively correlated with Anxiety (*r* = −0.18, *p* < 0.001) and positively correlated with Chosen Music (*r* = 0.20, *p* < 0.001). Discharge was negatively correlated with Music Making (*r* = −0.17, *p* < 0.001).

### Qualitative Results

Although participants were given the opportunity to provide free text answers about each music behavior they had engaged in during the pandemic, the question receiving the most responses was the first, regarding listening to self-selected music. Listening to self-selected music was the most frequently reported behavior overall, and additional the second most frequently reported increased behavior. Text answers from this question were therefore chosen for analysis for purposes of the current paper. A total of 116 (69 identified as female, 43 as male, 1 as transgender and 1 as non-binary) participants between the ages of 19 and 77 (M = 41.8 SD = 12.5) provided text answers to this question. They comprised 16 nationalities, the largest cohort of which were citizens of Finland (38.3%), followed by Canada (20.9%) and the United States (15.7%). The subset of participants who provided qualitative answers were thus demographically quite representative of the survey participants as a whole. A greater majority of this subset of participants reported living with others (78.3%) rather than alone. While only 15.7% of these reported that their city or country was in a state of lockdown, 89.6% of this subset reported that they were engaging in social distancing and 66.1% reported working from home. Thus, this subset of participants was experiencing less restrictions on their daily lives based on government regulations, but were nonetheless behaviorally fairly similar to the participant group as a whole.

#### Method of Analysis

The purpose of the research and the research questions were used to guide the choice of analysis method (Blair, 2015). A directed approach to content analysis was chosen, as this approach is useful for extending current theoretical understanding to a more specific phenomenon, as in the current case of examining musical engagement and its use in affect regulation in the novel context of a global pandemic and lockdown circumstances (Hsieh and Shannon, 2005). Content analysis focused on the research questions as they related to music listening; that is, how participants’ music listening had changed as a result of the pandemic, how their current music listening behaviors, as well as the role of music listening in their management of negative psychological effects of the pandemic such as mood and emotion. Data were analyzed using NVivo qualitative data analysis software (QSR International, Versions 11 and 12, 2015/2018).

To develop the coding scheme, a subset of 50 randomly-selected responses were first openly coded by one of the authors using the *in vivo* method, in which the specific language used by participants is preserved in order to gain insight into what is participants perceive as significant (Charmaz, 2006; Saldaña, 2016). After this, themes within participants’ responses emerged which related to how much their music engagement had changed and the use of music to regulate affect, including particular strategies. Once these higher order categories were established the remaining data were coded by another author based on these categories.

Simultaneous coding was used when appropriate, especially in the cases where participants had provided detailed descriptions of how they engaged with listening to music. Simultaneous coding is particularly useful when the data can infer several meanings (Saldaña, 2016). For example, participants provided insight into how frequently they engaged with music compared to before the pandemic, as well as insights into how they were using it in mood regulation simultaneously in their open-ended response. Following initial coding, subcategory themes that emerged within each category were identified. Axial coding was used in order to establish higher level thematic categories in the data (Saldaña, 2016). After all data had been coded once by the first two authors, a third author repeated the coding process using established codes to further verify the themes and subcategories. Very few discrepancies within coding emerged, which were discussed by the authors and agreed upon once coding had finished.

#### Results of Thematic Analysis

Of 116 responses, 107 responses were suitable for further analysis; discarded answers were off-topic or did not address the question (e.g., describing a personal situation without mentioning music listening). Three major categories were established that describe: (1) How the pandemic influenced the frequency of music listening; (2) How music helped support psychological needs during the pandemic, and (3) What kind of music listening activities participants engaged with during the pandemic.

##### Frequency of musical engagement

The qualitative data provided more insight into how the pandemic influenced participants’ amount of musical engagement. The first three categories were related to the amount of musical engagement and were categorized based on whether the participant indicated high amounts of music use during the pandemic (“More Engaged”), less amounts of music use (“Less Engaged”), or no significant changes in music use since the pandemic (“No Change”). The fourth category, Change in Engagement, represents cases where the frequency of musical engagement was not necessarily affected, but rather *how* participants engaged with music had changed. Descriptions of these categories are in Table 2.

**TABLE 2:** Major themes which emerged from free text answers.

**Level of engagement (n cases)**	**Description**	**Themes**
More Engaged (45)	Reports high amount of music use and/or have adapted their music engagement during the lockdown	• General increase in music listening
		• Increase in certain media formats
		• Routine changes enable musical behavior
Less Engaged (21)	Less engaged with musical activities since the lockdown.	• Routine changes disable musical behavior
		• Replacing music listening with another activity
No Change (15)	No significant changes in musical engagement, including amount of music use or method of engagement	• No significant changes in routine
		• No significant changes in musical behavior, such as listening or playing
Change in Engagement (18)	Frequency of music engagement is generally the same, but the type or method of listening has changed.	• Selecting different genres
		• Use of different listening devices
		• Seeking out different listening methods (tactics)

Out of the 107 cases analyzed, high amounts of music use were the most frequently reported; 42% of cases indicated they were highly engaged with music during the pandemic. These cases comprise the “More Engaged” category, which was further subcategorized depending on whether the participant reported (1) increased musical engagement as a result of routine change, (2) a general increase in music listening or playing, and/or (3) an increase in certain formats (streaming live concerts, listening to physical records instead of streaming). Descriptions of the More Engaged category and related subcategories are in Table 3.

**TABLE 3:** Subcategories related to More Engaged.

**Subcategory (n cases)**	**Description**	**Themes**	**Example**
General Increase (20)	Overall increase in musical engagement. Includes	Exploring new music and old favorites	*“Had more free time available to explore music”*
	listening and playing/singing.	Listening to music instead of other activities	*“I have listened to fewer audiobooks and podcasts, more music that makes me happy.”*
		More playing music (instrument, singing)	
			*“I play almost every day my piano for my self and for neighbors. [sic]”*
Other Formats (18)	Increased use of different music media.	Using physical media (vinyl, CDs)	*“I am listening to my vinyl records a lot more, almost a daily basis. Before it was once or twice a week.”*
		Using different platforms to stream music such as social media platforms (Facebook, YouTube)	
			*“In social media I have listened some virtual gigs done by artists that I follow. [sic]”*
		Watching livestreams and/or old concerts	
			*“Less radio, more streaming.”*
Routine Change (17)	Changes in routine as a result of lockdown enables listening.	Listening while working from home	*“I play music for myself and the family a lot now, while we are all at home, whereas before it was for me.”*
		Influence of family or flatmates	
		More control over music selection and when they can listen	
			*“Working from home lets me listen to or play music almost whenever I want.”*

Less Engaged individuals accounted for 19.6% of the qualitative data cases. Unlike the More Engaged category, decreases in musical engagement were less varied. Two subcategories were created in order to reflect the most salient themes in the data. The first subcategory represents cases where routine changes resulted in a decrease in music engagement. The second subcategory reflects the cases where music listening was replaced with a different, non-musical activity. Subcategories and examples are in Table 4.

**TABLE 4:** Subcategories related to Less Engaged.

**Subcategory (n cases)**	**Description**	**Themes**	**Example**
Routine Change (18)	Changes in routine negatively influenced music listening engagement	No longer commuting, traveling	*“I usually listened music during traveling to my work, and now I stay at home, so there is no traveling time [sic].”*
		Other people in the living space make music listening more difficult	
			*“*…*I’m living with my family. This means*
		No longer going to the gym or walking, decreases music listening opportunities	*I’m more restricted with regards to when and what music I listen to.” “Used to listen to music more often while running. Running has come to a complete standstill now.”*
Replace (7)	Music listening activities replaced by different media	Listening to audiobooks or podcasts instead of music	*“I’ve started to listen to audiobooks more than before.”*
		Watching news or TV streaming services instead of music	*“Replacing music with more Netflix.”*

Cases belonging to the No Change category accounted for 14% of the qualitative data. These participants reported that their music listening behaviors had not changed in any significant way as a result of the pandemic; although they may have experienced changes in routine, such as working form home, this change had no influence on their listening behavior. Unlike the other frequency categories, No Change descriptions were homogenous and did not require further subcategorization. Examples of No Change codes include responses such as (P622): *I usually listen to music during breakfast and when I work from home. This hasn’t changed*; and (P220): *I am working from home. While I work I often listen to music. I have also played music myself. Both things have not changed since the lockdown in the country I live in.*

The last category related to frequency of music use was the Change in Engagement category. This category reflects cases where a change in amount of music listening was not necessarily mentioned; instead, the method of engagement had changed, reflecting how the individual adapted their listening behavior to their circumstances. Of the 18 cases coded in the Change in Engagement category, 66.6% of these cases (12 in total) also had codes in at least one of the More Engaged subcategories. It was possible for a case to have codes in both the Change in Engagement category and More Engaged cases, since some cases provided more detail in their responses than others. For example, one case includes the code: *Now I have more time to listen to music. So I use this opportunity. [sic]* This code was categorized under the More Engaged subcategory since it describes more frequent music listening but does not suggest a change in *how* the individual engages. On the other hand, some cases provided data that inferred greater level of musical engagement in general *as well as* a change in *how* they engaged with music. For example, the following case excerpt contains two codes (P448): “*I am listening to my vinyl records a lot more, almost a daily basis. Before it was once or twice a week. It is also giving me more opportunities to really engage with the music.*”

The first two sentences suggest they are using vinyl records more frequently than they did before the pandemic. The last sentence suggests they are engaging more frequently with music as a result of the lockdown giving them “*more opportunities to really engage with the music.*” This also complements their results in the quantitative data, since this participant responded *Yes* to the question “Has the way you engage with music on a regular basis changed as a result of the coronavirus pandemic?” These participants also indicated that they listen to music “much more” compared to before the coronavirus pandemic in the corresponding survey question. Examples of this category are provided in Table 5.

**TABLE 5:** Subcategories related to Change in Engagement.

**Subcategory (n cases)**	**Description**	**Themes**	**Example**
Format and Devices (15)	Music listening devices and formats have changed in order to adapt to lockdown circumstances	Listening with different devices	*“I have music on my headset while working.”*
		Increased engagement with livestreaming media	*“Watching live online performances.”*
			*“No live concerts only records and streaming concerts and Youtube videos.”*
Genre and Preference (4)	Music preferences are noticeably different.	More interest in different genres	*“[I] always had ambient, electronic or deep house music on, in the background while I worked in the computer*…*Nowadays I mostly listen to jazz while relaxing at home. [sic]”*
	Choice of what to listen to has changed during lockdown	Selecting music with features that are more compatible with lockdown environment	
			*“Music is getting heavier.”*
			“*The music I choose seems to be less discreet than usual (since there is more noise to overcome).”*

##### Musical activity

The Musical Activity category sheds light on how participants engaged in music listening and playing for their leisure during the lockdown, as well as the ways music supported other, non-music related activities.

Two subcategories were established: Music for Activity and Music as Activity. Codes in the Music for Activity subcategory describe how music functioned in the background in order to support daily routines related to working from home, house chores and physical exercise. On the other hand, the category Music as Activity includes codes that describe music listening or playing as the *primary* activity, and includes activities such as focused music listening, watching live streams or recordings of old concerts, and playing music for family and neighbors.

The Musical Activity category was established since it sheds light on how music listening helped individuals adapt to their change in routine. While the influence of routine change is also highlighted in the frequency categories above, they do not adequately represent on their own the ways in which individuals adapted their listening behavior during the pandemic. One particularly intriguing example is the use of livestreams to replace the concert-going experience. This not only reflects how the individual adapts their listening behavior, but sheds light on the role of social media sites such as Facebook and (especially) YouTube and their function as music listening platforms in the context of the pandemic.

##### Music for psychological health and well-being

Overall, there were 26 cases in the qualitative data set describing how music influenced psychological health during the pandemic. These cases provided insight into the diverse ways in which music was used in order to achieve or support a particular affective state, as well as cases where music listening did not have its intended effect. Four subcategories emerged in the data (see Table 6). The first three describe the ways that music supported psychological health and well-being, and include: (1) music listening to raise or lower arousal (Increase Energy); (2) music listening in order to achieve or maintain a positive affective state (Create and Maintain); and (3) selecting music that evokes memories from the listener’s past (Nostalgia). Examples of these types of responses appear in Table 6.

**TABLE 6:** Subcategories related to mood regulation.

**Subcategory (n cases)**	**Description**	**Themes**	**Example**
Influence Energy (9)	Music listening in order to influence arousal	Music listening helps to relax, relieve tension	*“I listen relaxing music because I feel tension in my body [sic].”*
		Music helps increase energy levels	*“I like to put on upbeat songs to get me going.”*
Create and Maintain (9)	Music listening helps create or maintain desired affective states	Selecting music according to current mood	“*I chose music according to my mood.”*
		Music listening as an emotional outlet	*“I have listened to fewer audiobooks and podcasts, more music that makes me happy.”*
		Using music to distract from negative thoughts or emotions	
Nostalgia (4)	Listening to music associated with past memories.	Selecting music that was popular during their childhood	*“I have recently chosen to listen to music form my childhood more often*… *I wanted to be alone with the memories of the music.”*
		Curating playlists that remind them of youth	*“I have been making playlists that remind me of my younger days.”*
Negative Outcomes (7)	Music engagement had a negative influence on affective state	Music evokes feelings of unease, anxiety	*“*…*I’ve found that music with a fast beat or high intensity is more disturbing to me [now] than it has been in the past. I think it triggers anxiety in me?”*
		Inability to enjoy certain music, avoids particular genres, artists, etc.	
			*“No rock music at the moment. Can’t tolerate it at this time. It is usually my ‘go to.”*
		Engaging in musical activities such as playing elicits strong negative emotions	
			*“We have virtual sermons, but I do not singalong with the songs at home via internet.*
			*It does not feel the same and feels stupid to sing alone at home.”*

There were six cases in the data that describe music as having a negative influence on the participant’s affective state. These cases were subcategorized under Negative Outcomes, and describe how music listening had an undesired effect, such as triggering anxiety. Codes that describe avoiding certain music, including the genres or artists, that the participant preferred before the pandemic are also included in this subcategory.

Although this represents too small a sample for meaningful statistical comparison, a brief examination of the data revealed that these participants have a notably higher mean score on the BAI (*M* = 44, *SD* = 10.79) than the remaining participant group as a whole (*M* = 30.65, *SD* = 9.26). A score of 36 or more is considered a severe level of anxiety and may indicate the need for medical intervention. Individually, this group of participants’ BAI scores ranged from 30 to 60, with four out of the six participants meeting criteria for severe anxiety.

## Discussion

The current research represents an exploratory survey study of the role of music in everyday life during the first wave of the 2020 coronavirus pandemic. Results indicated that participants were engaging with music in a variety of ways, and furthermore that their engagement with music had changed in a variety of ways as a result of the pandemic. Those who reported changes in their frequency of musical engagement more often reported that their engagement increased.

The behavior reported as most affected by the pandemic was listening to self-selected music; 38.7% reported doing this more than before the pandemic, while only 16.6% reported listening to self-selected music less frequently. This may partly be because this was the most common musical engagement behavior overall, which is unsurprising as more people listen to recorded music on a daily basis than play an instrument or regularly attend concerts.

Participants’ free text answers suggest that changes in listening to self-selected music was also due in part to changes in routine such as working from home, as music-listening choices can in many cases be made more freely at home than in a work environment. However, the lack of a regular commute represented a loss of opportunity for routine listening for some. Some participants also reported that loss of fitness or other physical activities that had previously been part of their routines resulted in listening to self-selected music less frequently. Similarly, the most frequently reported decrease was in listening to music on the radio or via a streaming service such as Spotify, which several participants reported was due to no longer listening to music on their commutes. Participants also reported dancing less often, which may reflect that dance is typically a part of specific social settings such as clubs or weddings, as dance is arguably a fundamentally social behavior (Laland et al., 2016). Listening to music selected by someone else implies a social context, and this, too, was reported to have decreased by some participants. In their free text responses, some participants reported replacing regular music listening with other activities such as listening to audiobooks or watching Netflix, which may reflect individual differences musical engagement in general.

The most frequently reported increase was in streaming live music performances, which almost certainly reflects an increase in both availability and awareness of concert streaming as an opportunity for musical engagement; the former born out of necessity for artists to continue to work and the latter out of desire by audiences to experience live performances. Whether this increase in live streaming of concerts continues at some level after it is no longer necessitated by pandemic conditions remains to be seen and will require future investigation. It should be noted that, despite the fact that listening to the radio or streaming services was behavior most frequently reported to have decreased (18.9%), a greater number of participants (24%) actually reported that they have started doing this more often, possibly for similar reasons related to working from home. Individual differences of personality, interest in music, and use of music in mood and affect regulation may also have played a role in determining musical engagement, however, further research is required to investigate this question in depth. It is also important to note that, on all behaviors except for listening to self-selected music, the majority participants indicated no change in their behavior as a result of the pandemic, suggesting that musical engagement may, for many, have been one of few aspects of daily life that remained unaffected by the pandemic.

Analysis of the relationships between musical engagement, participants’ use of music for mood regulation, and participants’ anxiety and worry about the coronavirus situation revealed several interesting patterns. Participants who favored Cognitive Management strategies such as Mental Work (using music to think through problems) and Diversion (using music to distract from negative thoughts) tended to have higher levels of anxiety and be more worried about the effects of the pandemic as far as contagion within their families and communities. The high loading of Strong Sensation onto this factor may in part be explained by its correlation with Music Performances; Strong Sensation in this case may have been more related to seeking powerful esthetic experiences than to feeling strong emotions, as esthetic judgments involve higher levels of cognition (Brattico and Pearce, 2013) and may therefore relate more two the other cognition-based strategies which loaded onto this factor.

The negative correlation between anxiety scores and Arousal Management, that is, using music for Entertainment (e.g., making boring tasks more enjoyable) and Revival (relieving fatigue), may suggest that these mood regulation strategies were more effective at managing anxiety than others for participants who used these strategies; that is, although the direction of causality is not clear, it is possible that using these listening strategies resulted in lower anxiety for these participants. One explanation for this is that these strategies seem to target achieving an optimal level of physiological arousal, which may relate to anxiety insomuch as anxiety is associated with heightened arousal levels. However, these results may also suggest that these strategies were chosen by those who were already less anxious, while participants experiencing greater levels of anxiety were more likely to turn to other strategies to manage their mood. Further research is necessary to clarify this.

Analysis of participants’ free text responses about their music listening provided further insights into the role of music in regulation mood and emotion for some during the pandemic, corroborating the finding that some participants were using music to achieve optimal levels of arousal. Several participants also described using Diversion, specifically choosing music with a positive valence to distract from a negative mood, or choosing to listen to music rather than the news in order to avoid becoming anxious or upset.

A theme that emerged from the qualitative analysis not covered by the quantitative questionnaires was that of nostalgia. Nostalgia is a complex emotion that can be associated with positive, negative or mixed emotions. When evoked by music, nostalgia is moderated by individual differences such as personality, mood state, and the relationship of a given piece of music with individual, autobiographical memories (Barrett et al., 2010). In line with these definitions, the three participants who described using music to evoke nostalgia did not provide details on how the music influenced their mood, or whether they had specific goals related to emotion regulation. While it is not clear from these findings whether this indicates the use of music vehicle for remembering the past during times of change and uncertainty, this is a possibility which further research could help clarify this in the future.

Another finding arising from participants’ free text responses requiring, which requires further investigation, is that of occasional negative responses to music. These participants described changes in how they responded emotionally to what previously was their preferred types of music (genres, artists). Musical features, such as lyrics, and particular acoustic properties made these participants more aware of their anxiety surrounding the pandemic. Although based on only six participants and therefore not adequate for drawing generalizations, it is still arguable that one of the most notable findings from the current study was the relationship between having reported such negative responses to music and having clinically high levels of anxiety. This was based on only a handful of participants and may therefore be a coincidence, however, it may also be that negative responses to music may be a warning sign of severe anxiety and need for mental health intervention in some. The role of music for people with or at risk for depression has recently begun to be explored in the literature; Garrido and Schubert (2015), for example, found that listening to sad music, while pleasurable for some, increased sadness in those at risk for depression. However, although music has been shown to decrease anxiety in medical (Wu et al., 2017) and therapeutic (Guétin et al., 2009) settings, the role of everyday music listening in people suffering from clinical anxiety disorders has received less attention. At the very least, these results indicate that more research should be done on this topic as soon as possible.

Overall, the results of the survey show that music continued to play a role in everyday life for people affected by the first wave of the coronavirus pandemic. For some, music seemed to played an increased role in the management of mood and emotion. Different people used different strategies in music listening for mood regulation, with an apparent distinction between uses focused on managing conscious thought and uses focused on managing energy and arousal. Correlations between these strategies and reported levels of anxiety merit further research into causal effects. As this data was collected during the first half of 2020, however, it is unknown to what degree these results generalize to explain musical engagement during the later stages of the pandemic, making further research necessary. The qualitative data was additionally limited by inconsistency in how much detail was provided by participants and the lack of opportunity for follow-up. As the current data includes mainly European and North American participants, further research is also necessary to clarify how the effects of the pandemic on musical engagement may be affected by differing cultural norms or socio-political contexts. Despite these limitations, however, the current results suggest music and an accessible and potentially effective resource for coping during the pandemic, corroborating previous work and revealing new directions for gaining a further understanding how music helps us to cope with crisis.

## Data Availability Statement

The raw data supporting the conclusions of this article will be made available by the authors, without undue reservation.

## Ethics Statement

Ethical review and approval was not required for the study on human participants in accordance with the local legislation and institutional requirements. The patients/participants provided their written informed consent to participate in this study.

## Author Contributions

EC, H-RP, and SS conceived of the study and all authors contributed to the development and dissemination of the survey. EC and PT designed and carried out the quantitative analysis. EC, JW, MB, DD, H-RP, and SS developed qualitative analysis. JW and EC performed initial coding. MB performed secondary coding. JW developed category and subcategory structure in discussion with the above authors. EC and JW wrote the manuscript with respect to quantitative and qualitative results respectively, with feedback from all authors. H-RP, PT, and SS provided senior research supervision.

## Conflict of Interest

The authors declare that the research was conducted in the absence of any commercial or financial relationships that could be construed as a potential conflict of interest.

## Footnotes

1 www.alchemer.eu
